# Atavism and Reproduction

**Published:** 1883-01

**Authors:** John N. Navin


### Art. VI.—ATAVISM AND REPRODUCTION.

BY JOHN N. NAVIN, V.S.

THE above kindred subjects have been pretty thoroughly discussed through agricultural papers and stock journals for many years past and lately in college journals. Many papers have been written pro and con, on both subjects by scientific men, while other articles were from men having no standing in the veterinary profession and unable to advance one argument based on anatomy or physiology in proof of their theories. In the vast bable of conflicting opinions upon the subject of reproduction, I find some of undoubted ability advocating a theory by means of which breeders of stock are able to control the sex by treatment of dams and sires prior to conception of the embryo. I gave the matter very little attention, until recently when I noticed a very sensible article on reproduction in the *Turf, Field and Farm*, and one on atavism in the *Live Stock Journal.*

I shall now quote the articles alluded to. The writer in the former says: "There is a point in embryoism beyond which mortal ken has not penetrated, a Rubicon which the most scientific mind cannot cross; there are secrets of reproduction which the creator has reserved to himself. To begin with, we cannot fix the sex."

The other gentleman, whose article appeared in the *Live Stock Journal*, says, in relation to the subject of atavism:

"The influence that the sire has upon the dam on her first impregnation, which appears to extend to her subsequent colts, is a difficult problem to solve. It has been held upon this theory, that if you breed a young thoroughbred mare to a cold-blooded horse, her subsequent produce will be ever afterwards adulterated with common blood from the nervous effect upon the dam from this first embrace."

The above theory I fully endorse except the influence by which the system of the dam is adulterated by the sire, and I

expect to prove that if a mare impregnated by a horse of a foreign breed aborts before it is vivified, her system will remain unadulterated by her impregnation, but the moment the embryo becomes vivified the mare's system receives its first step toward adulteration, and upon the full maturity of the offspring, whether delivered alive or posthumous, the dam becomes a full cross of the sire of foreign breed who impregnated her, and she becomes forever after incapable of breeding a thorougbred of her own stock by any sire, be he of her own lineage or foreign thoroughbred descent.

Common sense should dictate to us that neither part or parcel of the sire can enter into the nervous system of the female, and that the nervous effect upon a female in an act of copulation, is nothing more or less than the result of her being in heat at the time, and the mechanical contact of the organs of both cause the ejection of the natural secretions of both male and female, and each experiences a nervous sensation whether impregnation results from the act of coition or not.

If it be only a nervous shock, and the result of one act of copulation can change the thoroughbred nature of a mare, a subsequent embrace by a thoroughbred must exert a like change in favor of his breed, no part or parcel of either sire possibly entering the nervous system of the mare to remain there.

There is, however, another and most potent law of nature by which the entire system of the mare is changed into a cross of her first impregnation, but she is perhaps not invulnerable to a subsequent influence by another male of foreign breed whose cross may more fully adulterate her blood and estrange it from that of her ancestry, but the first adulteraton can never be eradicated from her system.

It may be well to inform the student that every animal in creation except those who impregnate themselves are impregnated through the medium of an ovum.

Fowls and fishes are provided with an ovary in which the ovum is produced and matured, its open end facing toward the organ through which impregnation is performed. A large number of ova are produced from the closed end of the sac and are found in different stages of development, from those scarcely observable to the naked eye to the fully matured egg near its open end. As the ovum next the open end develops, the female seeks the company of the male and one or two acts of copulation are sufficient to impregnate all the ova which have attained a certain degree of development, and though the male may be taken away and another replace him, the number impregnated will belong to the first male, and the residue will be a mixture of both, the first impregnator predominating.

It is quite different as regards our domestic quadrupeds however; an act of copulation and impregnation must precede the conception of the offspring.

Now we come to the point at which it is necessary to review the law of nature by which a female becomes a cross of the male who first impregnated her. To this end it is indispensible to know the anatomy and physiology of the male and female organs of generation.

*Female Organs of Reproduction.*—Every female whose ovum is fertilized within the body is provided with a sac called a uterus. There are also two ovaries, one on each side. These ovaries perform the same office in the quadruped that they do in fowls, the ovum being produced by them, but instead of its being ejected and hatched outside the body, the ovum must enter the uterus, there to be impregnated by the male.*

As soon as the ovum is ejected it is received in the grasp of the fimbria of the fallopian tubes, andpasses along its tubular centre into the uterus. During this slow process, and for a few days after the ovum enters the uterus the female is said to keep in heat, but her desire commences to cool soon after the ovum is received in the uterus.

The male deposit contains from one to several living animalculæ, which are the vivifying principle of the future embryo. When the animalculæ are deposited by the male, one or more

---

*The ovum or egg spoken of by the author is formed in the ovaries of the female. These organs correspond to the testicles of the male. They are two in number, one on either side of the uterus and a little above it. A projection from the uterus on each side reaches up to and embraces the ovary. This is the Fallopian *Tube*, and serves to convey the matured ova from the ovary to the uterus. This maturation of ova occurs at different intervals in different species, and when it does occur the animal is said to be in heat and intercourse with the male is liable to be followed by pregnancy.—ED.

are brought in the presence of the ovum and impregnation occurs.

*As to the sex.*—The possibility of its control by man, or treatment of the dam prior to or subsequent to conception, I deny. The dam has no control of her offspring from the fact that the sire furnishes the vivifying principle, and that as soon as the form and shape of the embryo presents the first rudiments of the anatomy of the future offspring, the spinal column, the brain, eyes, and the generative organs are the only organs visible, while very little change has taken place in the ovum, or female principle.

The natural inquiry would follow, had the animalcule been neuter when deposited by the male, and the influence of the dam fixed the sex, or had it, when furnished, been male or female, it being a living animacule when it entered the ovum? The most plausible conclusion is that the sex had been determined before it entered the ovum.

We know that mares, cows and sheep having twins or triplets by one act of copulation produce both genders. This is quite detrimental to the theory of the treatment of dams and sires. We also know that sows and bitches produce several of both sexes by one act of copulation.

*Atavism.*—What is the rule by which thoroughbred stock is determined? If the line of ancestry, or atavism is broken into by a male of a foreign breed when crossed on a maiden female of thoroughbred stock, the offspring is a half-breed, but the greater number of breedeıs have not been aware that she becomes a cross of her first impregnator. They do not understand how the adulteration of the female system is accomplished for life. To explain this I am carried back to the impregnation of the ovum and must more fully explain the ovum in the uterus.

The ovum, on entering the uterus is enveloped in a soft albuminous covering, which, as soon as impregnation takes place, attaches itself to the walls of the uterus, becomes the afterbirth or placenta, and serves two wonderful purposes of nature; the first of which is to hold the ovum and the future embryo in place. It adheres firmly to the walls of the uterus by a number of ciliated projections upon its external surface,

The second purpose of nature served by the placenta is that the umbilical cord of the offspring is firmly attached to it, and as the embryo grows larger so does the afterbirth, being supplied with blood by the uterus through the placental vessels. Indeed, so closely is the union between the afterbirth and uterus, that pulling the former violently away before a natural separation takes place, a dangerous hemorrhage of the latter is imminent, often resulting in the death of the mother. The reader will bear in mind that the embryo is attached to the afterbirth by the umbilical cord, and the afterbirth to the uterus by its connections, and as is well known by physiologists, no organ or embryo can exist independently of blood. So, here we have a circulating medium between the dam and her offspring. Let us now see how this law of nature is carried on by referring to the spermatozoa deposited by the sire.

The spermatozoa, as before remarked, when deposited by the male, is a living principle, having in it the vitality of the sire which augments as the embryo increases in size. No animate being can exist without blood, therefore as the embryo becomes larger the quantity of blood also increases, which is supplied by the mother. The growth of the afterbirth is promoted by blood from the mother.

*Vivification of the Embryo.*—The moment the embryo partakes of life the heart beats and the circulation of the blood commences.

We know that no animal can long exist except its blood comes in contact with the oxygen contained in the air. It is therefore certain that the blood of the living offspring is carried through its umbilical cord into the afterbirth and from it into the uterus, and carried with the dam's blood into her lungs, purified and mixed with it at every pulsation, and of the mingled blood some is retained by the dam and sufficient returned to the embryo to nourish it. Exchanged from offspring to mother, the first nucleus in the embryo being from the sire, mixing with that of the dam, no wonder she becomes a cross of her first impregnator and forever incapable of breeding a thoroughbred of her own purity. It is therefore true that the mother's system becomes a cross of her first impregnator, and that she entails it to her subsequent offspring by other sires for life.

One remarkable proof of atavism appeared sometime ago in the Knube Valley. A farmer had imported a hornless breed of cattle from Scotland, and like all new breeds spread over the neighborhood, but upon a full trial did not prove satisfactory, so were abolished. The last son of them was a bull calf, belonging to Mr. Wingate, the importer.

One cloudy night a neighbor informed Mr. Wingate that a black bear was among his cattle. Mr. Wingate, eager to destroy the bear, they being numerous at that time, forgetting his calf, and taking it for the bear, shot it, and for thirty years not a mulay could be found until three of Mr. Wingate's cows dropped hornless calves.

A veterinary surgeon of Scotland, named McGillivray, informs us that a Scotch gentleman, imported a quagga (a species of zebra,) and having a thoroughbred mare in an adjoining paddock to that in which the quagga had been kept, the gates dividing the paddocks were carelessly left open, the quagga impregnated the mare and she brought forth a striped colt. The colt and the quagga were promptly taken out of sight. The mare has since been bred for several successive years to many stallions of her own breed and every one of her colts showed the striping of the quagga. Many such instances are on record.

An idea prevails that the imagination of the mother controls the color of her offspring, and that things thrown upon them, such as blood, etc., mark the offspring of a woman ; that looking at an animal called a hare divides the upper lip of a child, but this is now known to be untrue.

In conclusion I confess my unbelief in the possibility of man, by any art or device, changing the sex of any animal by the treatment of its dam, whatever may be said of changing through the sire, and I do agree with the gentleman's remarks in the *Turf, Field and Farm.* "There is a point in embryoism beyond which mortal ken has not penetrated, a Rubicon which the most scientific imagination cannot cross." There are secrets in reproduction which can only be discovered by closer study and more extended experiments. At present we cannot determine the sex of any animal.

